# Superior electric storage on an amorphous perfluorinated polymer surface

**DOI:** 10.1038/srep22012

**Published:** 2016-02-23

**Authors:** Mikio Fukuhara, Tomoyuki Kuroda, Fumihiko Hasegawa, Takashi Sueyoshi

**Affiliations:** 1 New Industry Creation Hatchery Centre, Tohoku University, Sendai, 980-8579, Japan; 2 Waseda University Research Organization for Nano & Life Innovation, Green Device Laboratory, Tokyo, Japan; 3 JEOL Ltd, Akishima, Tokyo 196-8558, Japan

## Abstract

Amorphous perfluoroalkenyl vinyl ether polymer devices can store a remarkably powerful electric charge because their surface contains nanometre-sized cavities that are sensitive to the so-called quantum-size effect. With a work function of approximately 10 eV, the devices show a near-vertical line in the Nyquist diagram and a horizontal line near the −90° phase angle in the Bode diagram. Moreover, they have an integrated effect on the surface area for constant current discharging. This effect can be explained by the distributed constant electric circuit with a parallel assembly of nanometre-sized capacitors on a highly insulating polymer. The device can illuminate a red LED light for 3 ms after charging it with 1 mA at 10 V. Further gains might be attained by integrating polymer sheets with a micro-electro mechanical system.

Storage systems for electrical energy have been investigated extensively over the past three decades^1^,^2^,^3^,^4^,^5^. Efficient, high-energy-density electrical energy storage systems play an important role as a power source for devices such as batteries, fuel cells, and electric double-layer capacitors (EDLCs)^3^,^4^,^5^, and they provide an effective power supply from the grid. Compared to batteries, EDLCs are more powerful and possess a longer cycle-life. EDLCs have some practical disadvantages, however, such as narrow operating temperatures between 253–323 K, a voltage limitation of 3 V for nonaquatic electric solutions, and poor AC electric storage.

To develop solid supercapacitors without liquid solvents, we studied the capacitance of nanocrystalline de-alloyed Si-Al alloy ribbons^6^,^7^ and de-alloyed and anodic oxidized Ti-Ni-Si alloy ribbons^8^,^9^. These materials store AC electricity from 193–453 K with a voltage variation between 10–150 V and a DC capacitance of ~4.8 F (~52.8 kF/cm^3^). A common requirement for electric storage is a surface with nanometre-sized cavities and high electrical resistance. We assumed a surface structure consisting of a distributed constant-equivalent circuit of resistance and capacitance, and one that is analogous to the active carbons in EDLCs.

We report here about the superior electric storage on a polymer with high electrical resistance. We selected amorphous perfluoroalkenyl vinyl ether polymer (APVEP) film, CYTOP^TM^ (EGG, Asahi Glass) with nanometre clusters containing the organic siloxanes. The fluorine atom is well known as the most electronegative element in the contact electrification series^10^. Our results show that the insulating amorphous perfluorinated-polymer (APP) film with nanometre-sized cavities and a high work function is an ideal candidate for supercapacitors with potential applications in handheld electronic devices, transportation, and renewable energy storage for power grids.

The discharging behaviour under a constant current of 1 pA, 1nA, 1 μA, and 1 mA after charging at DC currents of 1 mA for 30 s is shown in Fig. 1a,b for the APP and the dielectric polyvinylidene fluoride (PVDF, KF Piezo, Kureha) polymers, respectively. Increments in the charging current prolong the discharging time for both films. To evaluate the additionality (i.e., the integration effect) of their respective film capacitances, we investigated the constant-current discharging behaviour as a function of the surface area of both films. Fig. 1c,d show the 3 nA constant-discharging behaviour for the APP and PVDF polymers, respectively, after 1 mA–10 V charging. The former shows an increase in the discharging area (i.e., the storage energy) by increasing the surface area, whereas the behaviour of the latter is almost the same despite the increase film area. This means that each capacitor on the APP film is connected with a parallel circuit, i.e., with integrated capacitance. Strictly speaking, the surface structure of the APP film consists of an electric distributed constant capacitor with low capacitance and high resistance (~48 TΩcm) (inset of Fig. 1a). Nevertheless, each capacitor on the PVDF film is connected by a series circuit, indicating the absence of an integration effect for electric storage. To provide visible evidence of the APP’s electric storage, we illuminated a red LED light. The device, which has a surface area of 600 mm^2^, lit the LED for 3 ms (inset of Fig. 1b) after the device was charged with 1 mA at 10 V. The fact that we were able to light the LED confirms the potential for electric storage.

A complex plane plot of the impedance data obtained from the APP film is shown in Fig. 2a. The polymer’s variation in impedance with frequency did not show the combined pattern of a line with a slope of 45°, which would associate it with the distributed resistance/capacitance in a porous electrode^11^ and a high-frequency semicircle (i.e., a series-passive layer). A near-vertical line in the Nyquist plot suggests evidence of a series-RC circuit as well as a graphene double-layer capacitor^12^. There were rapid increases in the imaginary impedance compared with the real impedance in the lower-frequency region (Fig. 2b). Moreover, the capacitive behaviour (near the −90° phase angle) all over the frequency region (Fig. 2c) is clear evidence of a series-RC circuit. Thus, APP films offer a near-ideal electric distributed constant structure (inset of Fig. 2c) for enhancing electric storage. However, the value of the series capacitance was 0.4 nF (178.8 nF/cm^3^, 88.9 μF/kg) at 1 mHz (Fig. 2d).

We investigated the surface characteristics to understand the microscopic origin of the APP’s higher electric storage. Figure 3a shows an atomic force microscope (AFM) image, and Fig. 3b shows the corresponding scanning Kelvin probe-force microscope (SKPM) image. These figures depict an uneven surface with a convexity of 28 nm in diameter and a concavity of 31 nm in length. The outside appearance of the surface bears a close resemblance to that of anodic oxidized amorphous TiO_2–x_ surfaces in previous papers^8^,^9^. The line profiles of the noncontact AFM reveal spots that are distributed on the uneven surface. These spots have a high work function *Ф,* averaging 10.13 eV (=5.65 (*Ф*_*Pt*_) + 4.48(*Ф*_*CPD*_). This means that they can store electric charges; the same is true for the TiO_2–x_ layer^8^. The value of the work function is higher than 9.1 eV and was obtained from an ultraviolet photoelectron spectroscopy measurement^13^. After an additional positive 20 V was applied for 60 s at the centre of the Si back surface (see Fig. S5 in Supplementary Information), we observed an electrostatic potential distribution (Fig. 3c) and the corresponding electrostatic potential profiles in four directions (L1, L2, L3 and L4) every 90° (Fig. 3d). The potential negatively increased to −76.93 eV (=5.65 + 71.28 eV) at the centre. This value demonstrates the remarkable electric-absorption effect of the APP under applied voltage.

We now turn to an explanation of the superior electric storage on the APP. The APP structure consists of PVDF main chains containing C = O and N-H radicles^14^ (Fig. 4a). The APP has rugged half-sphere surface (convex diameter of 2R, Fig. 4b) with nanometre-sized cavities. Notably, lattice contractions have been observed in nanometre-sized metallic particles, such as silver^15^, copper and platinum^16^, and gold^17^. Such contractions are due to the extreme confinement of the inner electrons—the so-called quantum-size effect^18^,^19^. This effect is interpreted by screening multiple inner-combined electrons for a strong positive-charge nucleus^20^. Analogously, we inferred that the superior electric storage on the uneven APP surface is due to the same quantum-size effect, which induces a relative increase in the combined electrons, resulting in fewer free outer electrons from the screening effect. The convex-diameter dependencies of the calculated electrostatic potential and the induced outer electron pressure of fluorine atoms surrounding the APP structure are presented in Fig. 4c by means of the Thomas-Fermi (TF)’s electronic screening theory (discussed at length in the Supplementary Information). The decrease in diameter increases the negative potential and the positive pressure. The calculated potential was consistent with the experimental data at a convexity of 14 nm in diameter. A microscopic schematic is provided in Fig. 4d. In sharp contrast to conventional EDLC, in which its electrode is parallel to the circuit, the electrically- negative convex portion of APVEP main chains and the electrically-positive concave one dominated by C=O and N-H radicles with permanent dipoles^21^ form many electric double layers (EDLs) perpendicular to the electrode (see Fig. S4 in Supplementary Information). This model shows that the more an EDL’s density increases (namely, the more convex-diameter decreases due to quantum-size effect), the more the electric storage increases. The uneven surface serves as a series of resistors for an insulation layer with tiny capacitors through the bulk, as shown in inset of Fig. 2c. Thus, in comparison with EDLCs, the APP device has some advantages, such as a wide operation temperature, higher charging/discharging voltage, larger integration, lighter weight, and prevention of *IV* drop at long distance from electrodes.

We demonstrated the storage potential for the APP given its nanometre-sized cavities on an uneven surface. The APP has a high work function, measured at 10.13 eV, and it succeeded in illuminating an LED. We inferred that the potential for electric storage is related to the quantum-size effect derived from the TF theory. The integration of the film with a micro-electro mechanical system is likely to provide even higher levels of charge storage for portable electric application.

## Methods

The APVEP film with nanometre-cluster was prepared by doping a 3-aminopropyl (triethoxy) silane-coupling reagent into the APP. Sheet specimens with a thickness of 15 μm were fabricated on the Si substrate by spin-coating. The specific density, glass-transition temperature, and water-adsorption rate was 2.03, 471 K, and 0.01%, respectively^22^. The devices were fabricated mechanically^8^.

Scanning Kelvin probe-force microscopy (NC-AFM, JSPM-5200, JEOL) based on the measurement of electrostatic force gradient was applied to measure the absolute electrical potential between the Pt-coated cantilever tip at 0 and 20 V and the APP surface as the work-function difference. The DC charging/discharging behaviour was analysed at 10 V, 1 pA~1 mA for ~300 s at room temperature, with a complex impedance between 1 mHz and 1 M Hz and 10 mV, using the galvanostatic charge/discharge on a potentiostat/galvanostat (SP-150, BioLogic Science). A red LED lump with standard power of 2 × 10^−4^ W was used to verify the electric storage.

## Additional Information

**How to cite this article**: Fukuhara, M. *et al.* Superior electric storage on an amorphous perfluorinated polymer surface. *Sci. Rep.* **6**, 22012; doi: 10.1038/srep22012 (2016).

## Supplementary Material

Supplementary Information

## Figures and Tables

**Figure 1:**
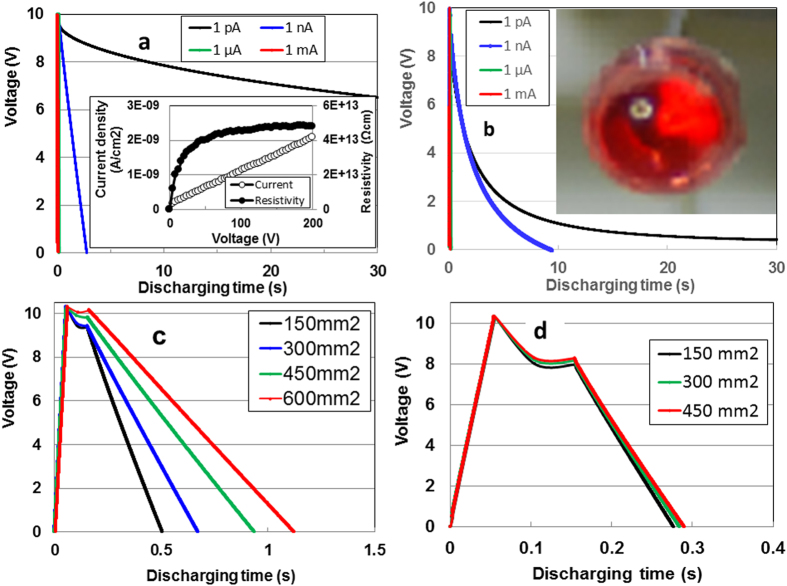
Discharging behaviour for the APP and PVDF devices after 1 mA–10 V charging. Constant-discharging (**a,c)** and integration effect on 3 nA-current discharging (**b,d**) for the APP and PVDF, respectively. Inset in (**a**): *I-V* and *R-V* characteristics, Inset in (**b**): illuminated LED light.

**Figure 2:**
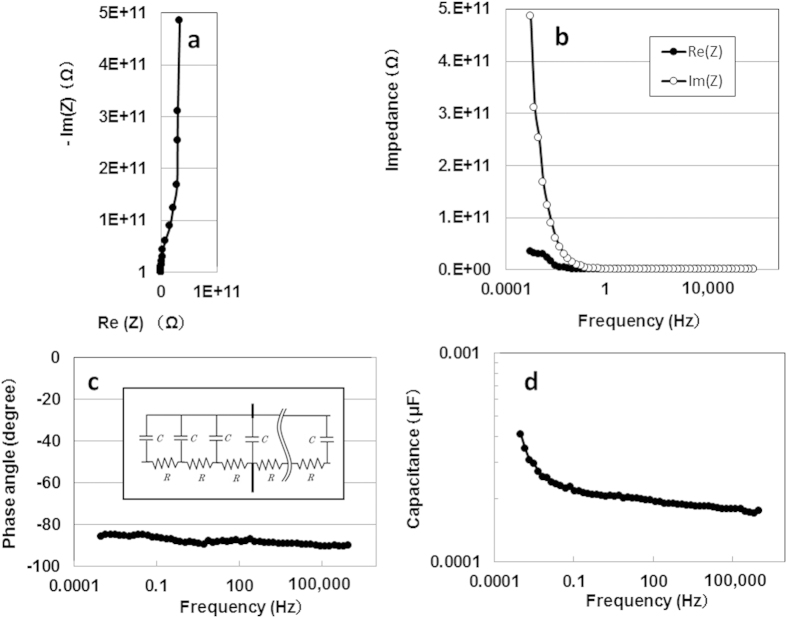
(**a**) Nyquist plot as a function of frequency for APP device. (**b**) Real and imaginary impedances. (**c**) Phase capacitance. (**d**) Series capacitance. Inset in (**c**): electric distributed constant-equivalent circuit of resistance and capacitance.

**Figure 3:**
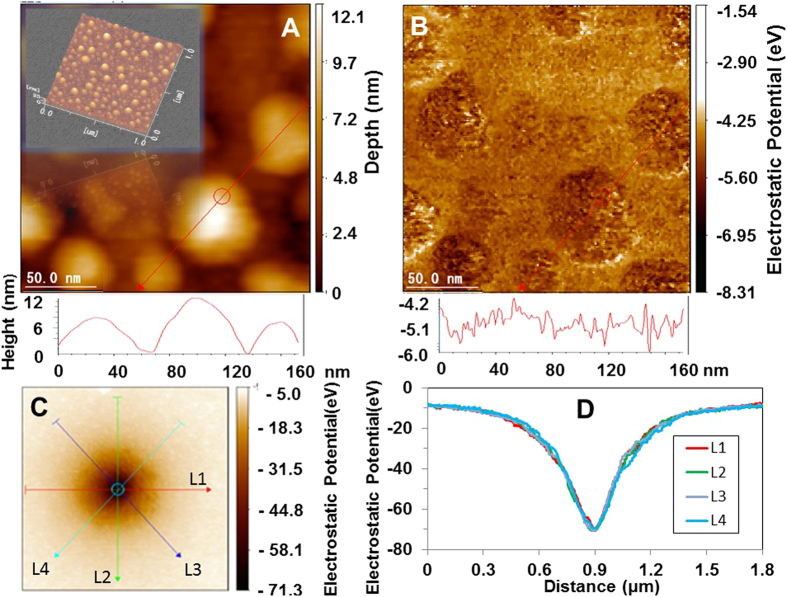
(**a**) AFM image of the APP surface. (**b**) Corresponding SKPM image. The lower profiles of (**a**,**b**) are the height from the valley bottom and the electrostatic potential for probe with 0 eV along red lines in upper images, respectively. (**c**) SKPM image. (**d**) Corresponding electrostatic profiles in four directions (L1, L2, L3 and L4) every 90° after applying an additional positive 20 V for 60 s at the centre of the Si back surface. Inset in (**a**): three-dimensional AFM image.

**Figure 4:**
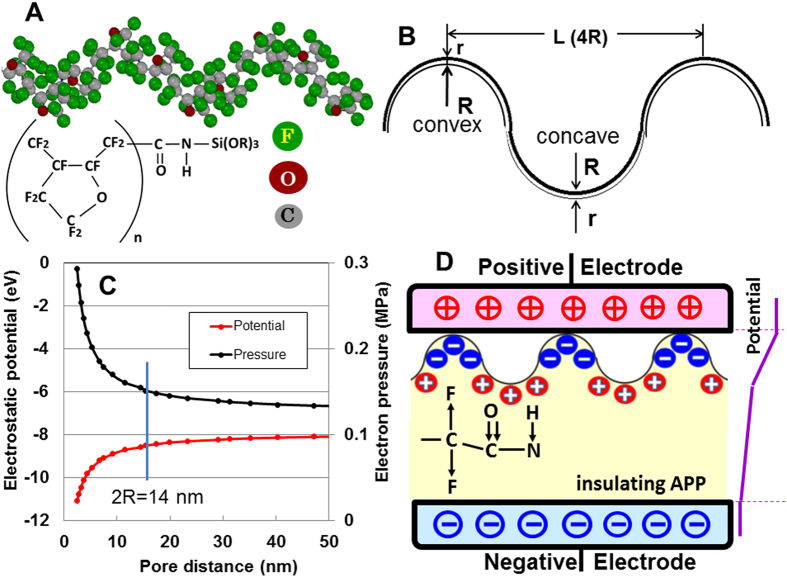
(**a**) Structural model of the APP with green fluorine, grey carbon, and red oxygen atoms, and possible structure with nanometer-sized clusters. (**b**) Schematic diagram for calculations based on the Thomas-Fermi statistical method. (**c**) Porous-distance dependence of the electron potential and pressure for the concave and convex portions. (**d**) Schematic representation of the microscopic electric energy storage used in this study.
