# Supplementary material for: Superior electric storage on an amorphous perfluorinated polymer surface
Source: Sci Rep. 2016 Feb 23;6:22012. doi: 10.1038/srep22012 (PMC4763234; doi:10.1038/srep22012)
Supplement: Supplementary Information [file srep22012-s1.pdf]

SUPPLEMENTARY INFORMATION

Superior electric storage on an amorphous perfluorinated polymer surface

Mikio Fukuhara [1,2] Tomoyuki Kuroda[1], Fumihiko Hasegawa[1] & Takashi Sueyoshi[3]

[1] New Industry Creation Hatchery Centre, Tohoku University, Sendai, Japan 980-8579

[2] Waseda University Research Organization for Nano & Life Innovation, Green Device Laboratory, Tokyo, Japan

[3] JEOL Ltd, Akishima, Tokyo 196-8558, Japan

PACS 77.22.-d, 77.84.-s, 77.90.+k, 78.55.Qr, 81.16.Pr

Supplementary Methods

Calculating the electrostatic potential of F on an uneven surface with nanometre-sized cavities using the Thomas-Fermi statistic method

Our interest lies in studying the electrostatic adsorption of fluorine atoms surrounding the APP with nanometre-sized cavities on an uneven surface with the quantum-size effect in view of the Thomas-Fermi (TF) electronic screening theory. The TF model has been applied to approximate calculations of potential fields and charge densities in elements as a function of lattice spacing. To the best of our knowledge, however, no detailed investigation has been conducted into the quantum-size effect for electric storage.

In general, nanoparticles with a particle size below 100 nm are characterised by a significant paucity in the ratio of chemical bonds in the particle surface. This suggests an increase in free electrons coming from the outer *s* and *p* subshells and resulting in a relative decrease in the inner subshells in nanoparticles. This physical picture explains the lattice expansion of nanoscale compound particles from the neutralization of the screening effect cause by the decreased binding-electron ratio[23]. By reverse analogy, then, we can calculate the electrostatic potential and the electronic pressure of the fluorine atoms surrounding the APP.

When we assume that the convex portion is almost a half-sphere, the ratio η of the topmost atomic layer volume V' to the half-sphere volume V increases as the half-sphere diminishes in size:

$$\eta = V'/V = 2\pi R^2 r/(4/6)\pi R^3 = 3r/R, \tag{S1}$$

where r and R are radii of the layer atom and the sphere, respectively. Because the outermost bonding of the surface atoms is missing, the missing electrons are apparently free. The ratio of free electrons to binding electrons increases as the size decreases, suggesting a relative decrease in the binding electrons. Because the free electrons squeeze into the inner binding-electron region in nanometre-sized metallic particles[24], the density ρ of the electrons associated with rigid bonding is calculated as follows:

$$\rho = \rho_0 (1+\eta), \tag{S2}$$

where ρ_0 is the electron density of the "bulk" atom.

The following relation derives the screening length $1/\lambda$ [25]:

$$\lambda^2 = 4\rho^{1/3}/a_o, \tag{S3}$$

where $a_o = h^2/me^2$ is the radius of the first Bohr orbit of a hydrogen atom. According to TF approximation, the screened Coulomb potential $\varphi(\mathrm{r})$ is written in the following form:

$$\varphi(\mathrm{r}) = qe^{-\lambda r}/r. \tag{S4}$$

When the screened Coulomb potential, the screening length, the electron density, and the atomic radius of the "bulk" and nanosphere atoms is φ_o, $1/\lambda_o$, ρ_o, r_o, and φ_1, $1/\lambda_1$, ρ_1, r_1,

respectively, we can solve

$$\varphi(r_o) = \varphi(r_1) \quad \text{(S5)}$$

for r_1 under an electrostatic equilibrium in potential at r_o and r_1, using the T-F table[23] $\varphi_0(x)$. We then consider an electronic contribution for the electric storage in terms of the bonding character of fluorine ions, using their electrostatic potential. Because outer electrons in fluorine ions are unsettled in the electronic structure with discrete permitted energies, several free electrons can move around the fluorine ions. According to ideal gas model, the pressure of the material arises almost entirely from the electrons, both because they outnumber the nuclei and because of their relatively small mass[26]. In electronic kinetic theory, the relation between electronic pressure P and the total number of electrons per unit volume n is given as follows:

$$P = \frac{1}{5}\left(\frac{3}{8\pi}\right)^{\frac{2}{3}}\frac{h^2}{m}n^{\frac{5}{3}}. \quad \text{(S6)}$$

The number n is related to effective potential energy U of the outer electrons as follows:

$$n = \frac{8\pi}{3h^3}(-2mU)^{\frac{3}{2}}. \quad \text{(S7)}$$

Eqs. (S6) and (S7) can then be combined as follows:

$$P = \frac{8\pi}{15h^3}m^{\frac{3}{2}}(-2U)^{\frac{5}{2}}, \quad \text{(S8)}$$

where h is a Plank constant and m is the mass of the electron.

From Eqs. (S5) and (S8), we can calculate the electrostatic potential and the induced outer electronic pressure of the fluorine atoms surrounding the APP. We used 0.119 nm as the ionic radius of fluorine in an F-C bond[27].

Supplementary Discussions

AC impedance analysis of poled APP and dielectric polyvinylidene fluoride polymers

We compared the AC impedance of the APP polymer poled at 8 kV for 180 s and the dielectric polyvinylidene fluoride (PVDF) polymer with the non-poled APP polymer shown in Fig. 2. The Nyquist diagram, the frequency dependence of real and imaginary impedances, the phase capacitance, and the parallel capacitance are shown at Figs. 1S**a**, 1S**b**, 1S**c** and 1S**d**, respectively. Both polymers behave similarly. Fig. 1S**a** shows the high-frequency region of the semi-circle in the Nyquist diagrams, and the simultaneous and rapid increase of real and imaginary impedances is shown in Fig. 1S**b.** The phase angle increased from -90° to 0° in the lower-frequency region, as shown in Fig. 1S**c**. This behaviour is clear evidence of a parallel-*RC* circuit, in contrast to the series-*RC* circuit of the non-poled APP film in Fig. 2. Thus, a higher voltage polarization for the APP film

appears to destroy the series-*RC* circuit on the surface. Indeed, the necessary and sufficient condition required for superior solid capacitors is to obtain the most resistant surface with a constant electrical distribution. Therefore, the non-poled APP device is a promising candidate for new electronic devices such as smart watches. Furthermore, the parallel capacitance in the PVDF shown in Fig. 1S**d** was 12 μF (178.8 nF/cm^3, 88.9 μF/kg) at 1 mHz, which is 20 times higher than those of both the non-poled and poled APPs. Judging from these results, it is clear that a superior piezo electric material does not always result in superior electric storage.

Comparison of the electric storage capability with other chloride films

To compare the electric storage capability of two chlorinated plastic-wrap films for the APP (1.99 GΩ), we selected polyvinylidene chloride (PVDC) film, Saran Wrap (Dow & Asahi Kasei, 1.33 GΩ), and Krewrap (Kureha, 0.89 GΩ). We measured the self-discharging behaviour of two kinds of PVDC film, both 600 mm^2 in size, after charging them 1 nA-10 V for 300 s using the circuit shown in Fig. S2. These results depend on a similarity in terms of the resistance of the specimen. The results are presented in Fig. S3, along with a curve of the APP. Both PVDC films rapidly decreased, compared with APP, indicating the latter's superiority for electric storage.

In conclusion, the APP device demonstrated superior electric storage as a result of the quantum-size effect on the nanometre-sized cavities along the surface with a work function of ca. 10 eV. The film is characterised by the surface structure with a constant distributed electric circuit, and it benefits from extremely high resistivity and extremely low water adsorption.

Model comparison with EDLC

Schematic electric-circuit configuration of the conventionary EDLC is presented at Fig. 4S**a**. It is characterised by a fractal structure which is composed of a distributed constant equivalent circuit of active carbon (R) and electrolyte (C)[6] (Fig. 4S**b**). The electrode of the EDLC is parallel to the circuit. On the other hand, the electrode of the APP device used in this study is perpendicular to the circuit, as shown in Fig. 4**d**.

Supplementary I-V and SKPM measurements

The current-voltage (*I*-*V*) and resistivity-voltage (*R*-*V*) were measured with DC voltages between 0 and 200 V at a sweep rate of 1.24 V/s, using a precision source/measure unit (B2911A, Agilent). Application of an additional positive 20 V is carried out by using SKPM. The schematic view is presented at Fig. S5.

Supplementary Notes

References for online supporting material

23. Kittel, C. *Introduction to Solid State Physics*, 4th edition (Wiley, 1971), p. 279.

24. Fukuhara, M. Lattice expansion of nanoscale compound particles. *Phys. Lett. A.* **313**, 427-430 (2003).

25. Condon, E. U. & Odabaşi, H. *Atomic Structure* (Cambridge University Press, London, 1980), p. 454.

26. Hamann, S. H. *Physico-Chemical Effects of Pressure* (Butter Worths Scientific, London, 1957), p. 59.

27. Shannon, R. D. Revised effective ionic radii and systematic studies of interatomic distances in halides and chalcogenides. *Acta Cryst*. **A32**, 751-767 (1976).

Supplementary Figure Legends

Fig. S1 **a, e,** Nyquist plots as function of frequency for poled APP and PVDF devices, respectively. **b, f,** Real and imaginary impedances. **c, g,** Phase angle. **d, h,** Series capacitance.

Fig. S2 Measuring system for self-discharging behaviour.

Fig. S3 Self-discharging behaviour for the fluorinated APP and the chlorinated Saranwrap and Krewrap films after a 1 nA-10 V charge for 300 s.

Fig. S4 **a**, Schematic electric-circuit configuration of EDLC. **b**, The distributed constant equivalent circuit for EDLC.

Fig. S5 Schematic view of SKPM measurement for application of an additional positive 20 V.

Supplementary Figures

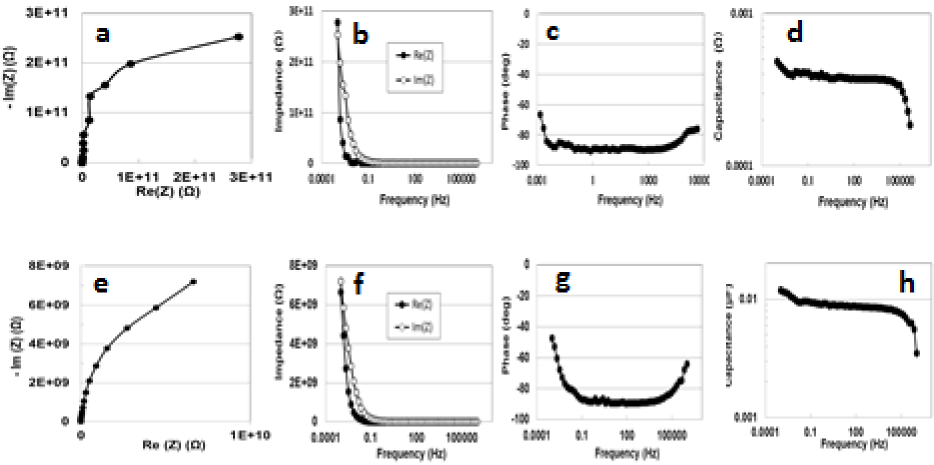

Fig. S1

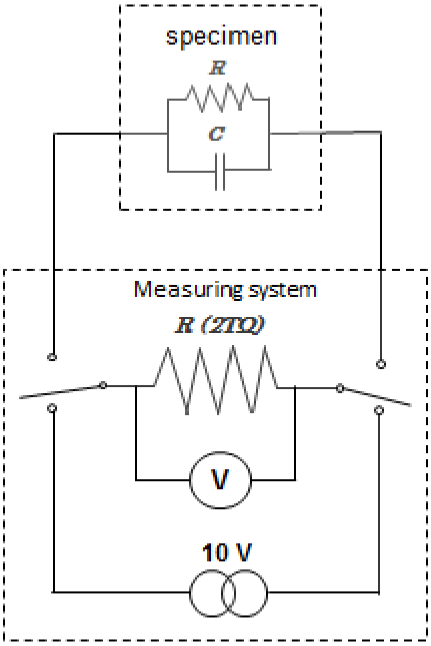

Fig. S2

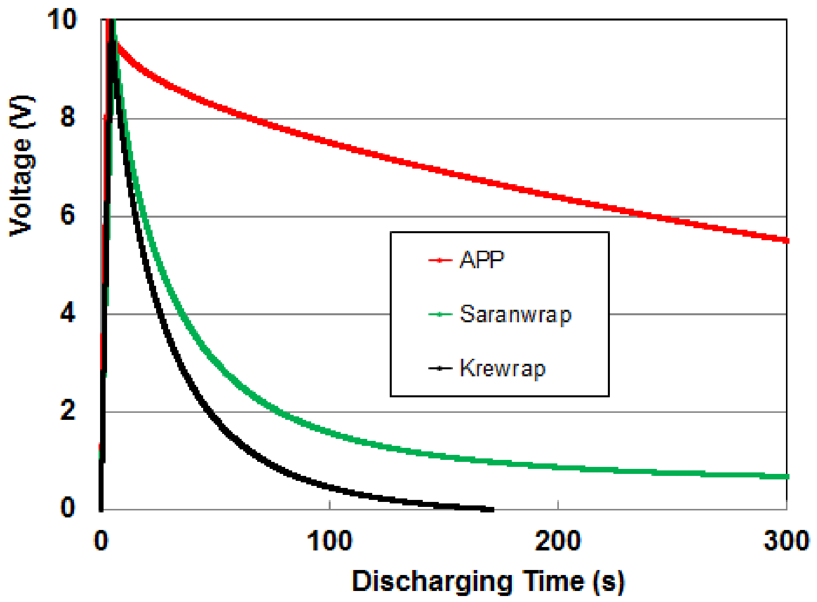

10
8
6
4
2
0
Voltage (V)
0
100
200
300
Discharging Time (s)
APP
Saranwrap
Krewrap

Fig. S3

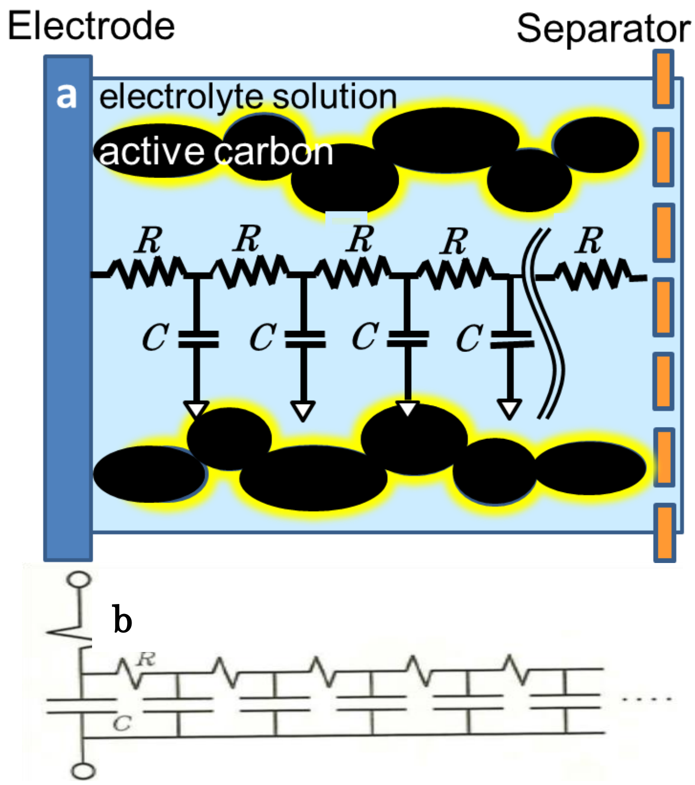

Electrode
Separator
a
electrolyte solution
active carbon
R
C
b

Fig. S4

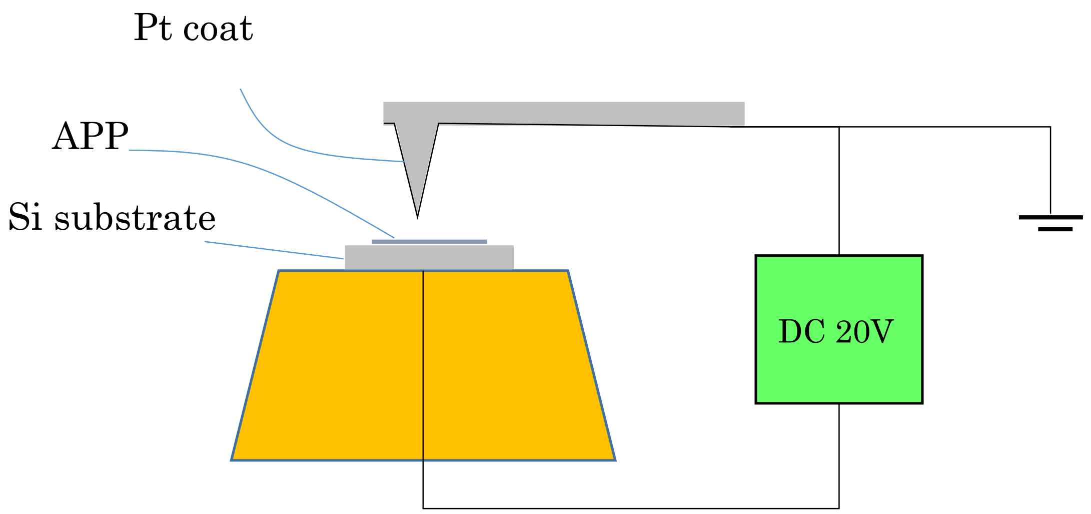

Fig. 5S
